# Bentonite-Based Functional Nanoclay Enhances Bacteriophage Therapy against Enteric Infections via Toxin Adsorption and Microbiome Recovery

**DOI:** 10.34133/bmr.0310

**Published:** 2026-01-29

**Authors:** Md Shohel Rana, Md Shamsuzzaman, Joo Hun Shin, You-Jeong Lee, Beoul Kim, Min-Goo Seo, Sung Man Seo, Sa-Hyun Kim, Je Chul Lee, Jungmin Kim, Shukho Kim

**Affiliations:** ^1^ Department of Biomedical Sciences, The Graduate School, Kyungpook National University, Daegu 41944, Republic of Korea.; ^2^ College of Veterinary Medicine & Institute for Veterinary Biomedical Science, Kyungpook National University, Daegu 41566, Republic of Korea.; ^3^ Advanced Geo-Materials Research Department, Pohang Branch, Korea Institute of Geoscience and Mineral Resources, Pohang 37559, Republic of Korea.; ^4^ BK21 FOUR KNU Creative BioResearch Group, School of Life Science, Kyungpook National University, Daegu 41566, Republic of Korea.; ^5^ Department of Microbiology, School of Medicine, Kyungpook National University, Daegu 41944, Republic of Korea.; ^6^ Untreatable Infectious Disease Institute, Kyungpook National University, Daegu 41944, Republic of Korea.

## Abstract

Diarrheal infections caused by antibiotic-resistant *Escherichia coli* pose a serious threat to human and animal health, driving the need for innovative therapeutic strategies. This study introduces a dual-action strategy that integrates bacteriophage EC.W2-6 with bentonite to enhance bacterial clearance and macromolecular toxin removal. Phage EC.W2-6 demonstrated high specificity against enterotoxigenic *E. coli* (ETEC) H10407, achieving nearly 100% adsorption to host cells within 15 min and a moderate burst size of approximately 80 plaque-forming units per infected cell. Bentonite exhibited substantial dose-dependent binding of ETEC-secreted proteins and outer membrane vesicles (OMVs), with the 30-g treatment showing the highest efficiency. Nanoparticle tracking analysis confirmed a 3.56-fold reduction in OMVs at 5 g bentonite and near-complete removal at 30 g. Physicochemical analysis indicated a stabilizing effect of bentonite, showing that bentonite–phage association partially neutralized the phage surface charge (from −34.2 to −13.4 mV), forming a more stable colloidal complex with an approximately 2-fold decrease in colloidal size. In a murine diarrheal model, single therapy with either EC.W2-6 (multiplicity of infection = 0.1) or 8% bentonite conferred 60% survival, whereas combination treatment provided 100% protection with a synergistic effect. Microbiome analysis revealed that dual therapy restored gut microbial diversity and suppressed *Proteobacteria* expansion, closely resembling healthy controls. These findings highlight the therapeutic potential of combining bentonite with phage therapy as an integrated macromolecular intervention against ETEC-induced diarrhea and intestinal dysbiosis.

## Introduction

Diarrheal disease caused by *Escherichia coli* is a major global health concern, affecting both human and animal populations. Among the various pathotypes of diarrheagenic *E. coli*, enterotoxigenic *E. coli* (ETEC) is a leading cause of infectious diarrhea, responsible for over 200 million cases annually in humans and substantial economic losses in the livestock industry [1–3]. ETEC colonizes the small intestine and induces infection by producing 2 major enterotoxins: heat-labile toxin (LT) and heat-stable toxin (ST). LT, an AB₅ toxin, binds to ganglioside GM1 receptors via its B-subunit, accumulating intracellular cyclic adenosine monophosphate (cAMP), while ST increases cyclic guanosine monophosphate (GMP) levels via guanylate cyclase activation. These signaling disruptions lead to ion secretion and water loss, triggering acute watery diarrhea [2,4]. The rising antibiotic resistance of ETEC strains has made treatment more challenging and motivates the development of alternative therapies [5].

One promising approach is bacteriophage (phage) therapy. These viruses specifically target bacteria by binding to surface components such as lipopolysaccharides (LPS) or outer membrane proteins like OmpA, enabling the selective elimination of harmful bacteria while preserving the beneficial host gut microbiota [6,7]. For instance, phages from the *Podoviridae* family utilize their icosahedral capsid and short tail fibers to bind specific receptors, enabling localized degradation of antibiotic-resistant strains [8,9]. Despite their precision, phage-based therapies face 2 critical challenges in the gastrointestinal environment. Firstly, acidic gastric conditions can inactivate phages before they reach the intestine [10]. Secondly, and more critically in gram-negative infections, bacterial lysis may lead to the sudden release of high concentrations of endotoxin (lipopolysaccharide [LPS]) and outer membrane vesicles (OMVs), potentially triggering severe inflammatory responses even after bacterial clearance, a phenomenon known as the “Endotoxin Dilemma” [10]. These intrinsic safety and delivery limitations of lytic agents necessitate cointerventions that simultaneously promote pathogen clearance and mitigate toxin release.

Bentonite, a natural clay mineral primarily composed of montmorillonite, has shown promise in biomedical applications. Its high surface area and layered aluminosilicate framework promote electrostatic adsorption of biological macromolecules like LPS, LT, and OMVs [11,12]. This binding can reduce toxin activity and limit inflammatory damage. Previous studies have shown that bentonite’s negatively charged surfaces can effectively immobilize the positively charged cationic sites of ETEC toxins [13,14]. While its toxin-adsorbing potential is well established, the integration of this effective macromolecular adsorbent with phage therapy to create a dual-action safety cointervention integrating bacterial lysis and toxin detoxification remains largely underexplored.

Although phage therapy has been investigated against ETEC and other enteric pathogens, existing approaches primarily focus on bacterial removal and do not adequately address post-lysis inflammatory toxicity or phage instability in gastric environments [10,15–17]. Likewise, bentonite and similar aluminosilicate adsorbents have been independently evaluated for toxin sequestration, yet their use has been limited to passive binding applications in livestock and human gut detoxification settings without integration into active antimicrobial strategies [11–14]. To the best of our knowledge, no study has combined a lytic phage with a macromolecular adsorbent to simultaneously (a) lyse ETEC, (b) neutralize released endotoxins/OMVs, and (c) protect the phage during gastrointestinal transit. This therapeutic convergence directly targets 2 major unresolved barriers in phage therapy for gram-negative infections: endotoxin surges following bacterial lysis and rapid phage inactivation under acidic conditions.

**Fig. 1.**
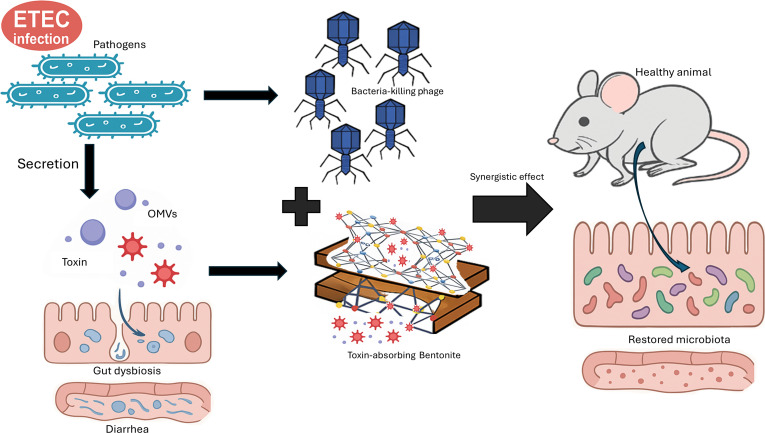
Schematic illustration of the dual-action therapeutic strategy. Phage EC.W2-6 specifically lyses ETEC bacteria, while bentonite adsorbs secreted enterotoxins and OMVs. This combination reduces pathogen load and toxin-induced gut damage, promoting microbiota restoration and resolving diarrhea.

In this study, we present a dual-action therapeutic strategy that combines the bacteriolytic specificity of phage EC.W2-6 (*Podoviridae* family) with the macromolecular adsorption capacity of bentonite. We hypothesize that this integrated approach will not only eliminate ETEC populations and enhance phage delivery through the protective excipient effect of bentonite, but also neutralize toxin-associated pathology by scavenging LPS and OMVs released during phage-mediated lysis. This dual mechanism is expected to promote more rapid and stable restoration of the gut microbiota. To test this, we (a) characterized EC.W2-6’s lytic activity against antibiotic-resistant ETEC; (b) quantified bentonite’s adsorption of LT, LPS, and OMVs through biophysical assays; (c) evaluated the therapeutic efficacy of this combination in a murine diarrheal model; and (d) analyzed gut microbiota to determine whether combination therapy promotes microbial restoration and mitigates dysbiosis. Together, these investigations offer a comprehensive macromolecular approach for treating ETEC infections by integrating biological and mineral-based interventions (Fig. 1).

## Materials and Methods

### Bacterial strain, culture conditions, and phage preparation

ETEC H10407 (O78:H11, LT/ST) was cultured on 5% sheep blood agar (Thermo Fisher Scientific) at 37 °C for 24 h. A single colony was inoculated into lysogeny broth (LB; BD Difco) and incubated at 37 °C with shaking at 150 rpm until reaching early exponential phase (OD₆₀₀ = 0.4 to 0.5). Phage EC.W2-6, previously isolated from sewage water, was propagated using ETEC H10407 as the host [6]. For amplification, H10407 cultures were infected during early exponential growth and incubated overnight at 30 °C. Cultures were then centrifuged at 8,000 × *g* for 10 min, and the supernatants were filtered through 0.22-μm polyvinylidene fluoride (PVDF) membranes (Millipore). Phage titers were determined using the double-layer agar method [15] and stored at −70 °C in LB broth supplemented with 15% (v/v) glycerol (Sigma-Aldrich).

### Phage lytic activity assay

Lytic activity was evaluated by spot assay as described previously [15]. To prepare the bacterial lawn, 100 μl of a stationary-phase bacterial culture was mixed with 10 ml of 0.75% soft agar and overlaid onto a BHA agar plate. After solidification, 15 μl of phage suspension (10^7^ to 10^8^ plaque-forming units [PFU]/ml) was spotted onto the bacterial lawn. Plates were air-dried and incubated overnight at 37 °C. Bacterial susceptibility was determined by observing the formation of clear lysis zones. To assess host range, the same procedure was applied to ETEC strains H10407-P, PB176, E8775, and E9034/A, as well as the nonpathogenic reference strain ATCC 25922.

### Adsorption rate and one-step growth curve

The adsorption test and replication profile of phage EC.W2-6 were determined using a modified protocol [16]. ETEC H10407 was cultured in LB to mid-exponential phase and infected with phage EC.W2-6 at a multiplicity of infection (MOI) of 0.0001. The mixture was incubated at room temperature, and aliquots were collected at 0, 1, 3, 5, 10, 15, and 20 min post-infection. To quantify unabsorbed phages, samples were centrifuged at 8,000 × *g* for 5 min, and the supernatants were analyzed using a double-layer plaque assay.

The one-step growth curve was used to determine the burst size and latent period. H10407 cells were harvested by centrifugation and resuspended in 1 ml of fresh LB to reach approximately 5 × 10^9^ CFU/ml. Phage EC.W2-6 was added at an MOI of 0.0001 and incubated at 4 °C for 30 min to allow synchronized adsorption. Following centrifugation at 12,000 × *g* for 5 min, the pellet was resuspended in 10 ml of fresh LB and incubated at 37 °C. Samples were collected at 5-min intervals over a 45 min period, serially diluted, and analyzed for PFU. All experiments were performed in triplicate.

### Phage thermal and pH stability

Thermal stability was tested by incubating phage suspensions (10^8^ PFU/ml) at various temperatures (4 to 80 °C) for 1 h. For pH sensitivity, phages were exposed to LB broth adjusted to pH 2 to 10 and incubated at 37 °C for 4 h. Residual phage activity was measured using the double-layer agar assay [17].

### Phage stability under simulated intestinal conditions

Phage stability was tested in simulated intestinal fluid containing 0.85% NaCl, 0.3% bile salts, and 1 mg/ml pancreatin (pH 8.0). EC.W2-6 (~10^8^ PFU/ml) was incubated at 37 °C for 6 h under 2 conditions: (a) phage alone and (b) phage with 8% (w/v) bentonite. Samples were taken at 0, 3, and 6 h. Each sample was diluted in SM buffer, vortexed to disperse any bentonite–phage complexes and titrated by the double-layer agar plaque assay. Survival (%) was calculated as (*N*/*N*₀) × 100, where *N*₀ and *N* represent PFU/ml at 0 and 6 h, respectively [18].

### Phage stability in bentonite at different pH levels

Bentonite (Korea Institute of Geoscience and Mineral Resources, South Korea) was prepared as 1%, 2%, 4%, and 8% (w/v) solutions in phosphate-buffered saline (PBS), vortexed for 5 min to ensure uniform dispersion, and mixed with phage EC.W2-6 (10^8^ PFU/ml) at a 1:1 (v/v) ratio. Phage–bentonite mixtures were incubated at 37 °C for 4 h at varying pH values (2 to 10), and viable phages were enumerated by plaque assay [17].

### Adsorption of toxin and OMVs by bentonite

To evaluate adsorption efficiency, ETEC H10407 was cultured overnight in 0.5 l of LB broth, then centrifuged at 10,000 × *g* for 10 min at 4 °C to obtain the supernatant. Bentonite (5, 15, and 30 g) was individually mixed with 100 ml of the supernatant and incubated at room temperature for 1 h under gentle agitation. After incubation, the mixtures were passed through 0.22-μm vacuum filters (Millipore) to separate bentonite–toxin or bentonite–OMV complexes from the unbound components and then further filtered using 0.22-μm syringe filters (GVS, ABLUO) to eliminate any remaining particulates. The resulting filtrates were analyzed for whole protein contents using a NanoDrop spectrophotometer (Thermo Scientific) and for OMV measurement using nanoparticle tracking analysis (NTA; Malvern Panalytical).

To further confirm adsorption, the bentonite pellet was resuspended in 9 ml of PBS and treated with 1 ml glycine-HCl buffer (0.1 M, pH 2.5). The mixture was vortexed and agitated at room temperature for 1 h, then centrifuged at 5,000 × *g* for 5 min. The supernatant was concentrated using a 10-kDa Amicon (Merck Millipore) centrifugal filter and analyzed by sodium dodecyl sulfate polyacrylamide gel electrophoresis followed by Western blotting as previously described [2]. Proteins were transferred to PVDF membranes, blocked in 5% skim milk (in TBST), and probed with an anti-LT antibody (1:1,000, Abcam) and a horseradish peroxidase-conjugated secondary antibody (1:5,000, Santa Cruz Biotechnology). Chemiluminescent signals were visualized using ECL reagent (Thermo Scientific).

### Zeta potential and physicochemical characterization of phage–bentonite interaction

Phage EC.W2-6 was prepared at a concentration of $10^{8}\;\mathrm{PFU}/\mathrm{ml}$, and bentonite suspensions were prepared in PBS at concentrations of 1%, 2%, 4%, and 8% (w/v). Control groups included phage lysate EC.W2-6 alone and bentonite 8% alone, both prepared under the same conditions. A 5-ml aliquot of phage was mixed with an equal volume of the respective bentonite suspension, vortexed for 10 s, and incubated at room temperature for 1 h with gentle agitation. The mixture was then centrifuged at 5,000 × *g* for 10 min, and the supernatant was collected. To remove residual bentonite particles, the supernatant was filtered through a 0.22-μm syringe filter and then subjected to centrifugal filtration using a 50-kDa Amicon centrifugal filter (Merck Millipore) to exchange the solution to water as required by the zeta potential (ZP) measurement device to prevent damage. The filtered supernatant was analyzed for ZP using a Malvern Zetasizer V2.2 (Malvern Instruments).

### Endotoxin quantification

A log-phase *E. coli* culture was inoculated with isolated phages and incubated at 37 °C until complete bacterial lysis. The lysate was centrifuged at 5,000 × *g* for 10 min, followed by filtration through a 0.45-μm filter. Bentonite was weighed to achieve final concentrations of 2.5%, 5%, and 10% (w/v) in a total volume of 4 ml of PBS. Two milliliters of phage lysate (10^10^ PFU/ml) was added to the 2 ml of weighed bentonite and vortexed for 10 s. The suspension was incubated at room temperature for 60 min with gentle agitation. The mixture was then centrifuged at 5,000 × *g* for 10 min. The supernatant, containing the purified phage, was collected and filtered using a 0.22-μm syringe filter to remove residual clay particles. Endotoxin levels in the filtered supernatants were measured using the Pierce Chromogenic Endotoxin Quant Kit (Thermo Scientific) according to the manufacturer’s instruction.

### In vivo evaluation of therapeutic efficacy

#### Animals

Six-week-old female BALB/c mice were obtained from Hyochang Science Laboratory (Daegu, South Korea) and maintained under controlled conditions with ad libitum access to food and water. All animal procedures were approved by the Kyungpook National University Animal Care and Use Committee (KNU: 2024-0534).

#### LD₅₀ determination

ETEC H10407 cultures were grown to an OD₆₀₀ of 0.5, centrifuged, and resuspended in PBS. Serial dilutions ranging from 10^7^ to 10^10^ CFU/mouse were prepared. Five groups of mice (*n* = 4 per group) were orally administered 100 μl of each bacterial dilution, while a control group received PBS only. Mice were monitored daily for 7 days to assess survival and clinical symptoms, following established protocols for LD₅₀ determination in ETEC infection models [2,6].

#### Bentonite dose optimization

Bentonite powder was suspended in PBS to prepare 1%, 2%, 4%, 8%, and 16% (w/v) solutions and homogenized by vortexing. Mice (*n* = 4 per group) were orally infected with 100 μl of *E. coli* H10407 (10^8^ CFU/mouse), except for a control group that received PBS. Two hours post-infection, each group received 200 μl of the respective bentonite solution. Mice were observed for 7 days to assess treatment tolerability and monitor any dose-dependent adverse effects.

#### Treatment and infection response

Mice (*n* = 5 per group) were randomly assigned to 5 treatment groups: (a) PBS control, (b) *E. coli* infection only, (c) phage EC.W2-6 (MOI 0.1), (d) 8% bentonite, and (e) combination of EC.W2-6 and 8% bentonite. All groups, except the PBS control, were infected via oral gavage with 100 μl of ETEC H10407 (10^8^ CFU/mouse). Two hours later, each group received 200 μl of the assigned treatment via the same route. Fecal samples were collected to assess diarrheal symptoms. Clinical signs and survival were monitored daily for 7 days to assess therapeutic efficacy.

#### Sample collection and analysis

At the end of the 7-day observation period, all surviving mice were euthanized under anesthesia. Intestinal tissues were aseptically collected, rinsed with sterile PBS, and immediately stored at −80 °C for microbiome analysis. Microbiome profiling was performed via 16S rRNA gene sequencing at CJ Bioscience (Sejong-daero, Seoul, South Korea). The resulting data were then used to assess microbial diversity and taxonomic composition across treatment groups.

#### Statistical and bioinformatic analysis

Statistical analyses were performed using GraphPad Prism (Prism 10.2.2). Survival data were analyzed using the Kaplan–Meier method, and differences between groups were assessed with the log-rank (Mantel–Cox) test. Comparisons of ETEC H10407 toxin adsorption and release by bentonite were evaluated using Welch’s *t* test. All results are presented as mean ± standard error of the mean (SEM).

Advanced statistical analyses of microbiome data were performed in Python using the scikit-bio and statsmodels packages. Differences in overall microbial community composition (beta-diversity) were assessed using permutational multivariate analysis of variance (PERMANOVA) based on a Bray–Curtis distance matrix with 999 permutations. Differential abundance at both the phylum and family levels was evaluated using the nonparametric Kruskal–Wallis *H* test. When the Kruskal–Wallis test indicated significant group-level differences, pairwise comparisons were performed using Dunn’s post-hoc test, with all *P* values adjusted for multiple comparisons using the false discovery rate (FDR) method. Statistical significance for phylum-level comparisons was defined as *P*_adj_ < 0.05, while family-level analyses used a threshold of *Q* < 0.20 for the overall Kruskal–Wallis test. Pairwise differences were considered statistically significant when *P* < 0.05.

## Results and Discussion

### Lytic activity and adsorption of phage EC.W2-6

**Fig. 2.**
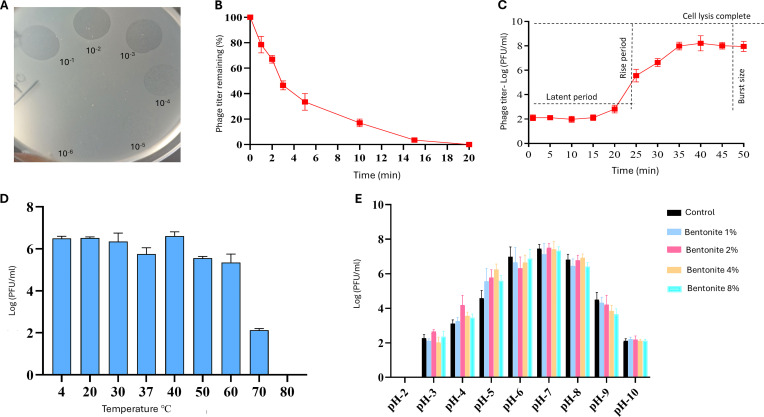
Antibacterial activity and physiological characterization of bacteriophage EC.W2-6 against *Escherichia coli* H10407. (A) Spot assay demonstrating lytic activity of EC.W2-6, with serial dilutions (10^−1^ to 10^−6^) applied to a bacterial lawn. (B) Adsorption kinetics at an MOI of 0.0001, showing 50% adsorption within 5 min and near-complete binding by 15 min. (C) One-step growth curve illustrating a latent period (~15 min), rise phase, and burst size of approximately 80 PFU/cell. (D) Thermal stability of EC.W2-6 following 1-h incubation at 4 to 80 °C, showing significant loss of infectivity above 60 °C. (E) pH stability of phage EC.W2-6 after 4-h exposure to pH 2 to 10, assessed in the absence (control) and presence of bentonite (1% to 8%). Maximal viability was maintained between pH 5 and 8. Bentonite notably improved phage viability under acidic conditions (especially pH 3 to 5), supporting its protective role in gastrointestinal environments. Data are shown as mean ± SD (*n* = 3).

Phage EC.W2-6 exhibited potent lytic activity against ETEC H10407, forming distinct, clear plaques across dilutions from 10^−1^ to 10^−6^ in spot assays (Fig. 2A). These findings confirm its efficient host recognition and strict lytic behavior, a desirable characteristic for therapeutic application [19]. Adsorption analysis showed that 50% of phages bound to host cells within 5 min, with near-complete adsorption by 15 min (Fig. 2B). This rapid attachment suggests a strong receptor-binding affinity, facilitating faster infection onset and efficient bacterial clearance. Such properties enhance EC.W2-6’s relevance for phage therapy, particularly against multidrug-resistant *E. coli* strains [6].

The host range of EC.W2-6 was further tested against multiple ETEC strains and reference bacteria. EC.W2-6 lysed H10407-P, PB176, H10407, and E8775 strains expressing LT and/or ST toxins and showed activity against the nonpathogenic reference ATCC 25922. In contrast, E9034/A exhibited semi-clear lysis, potentially due to surface structure variation or receptor inaccessibility (Table S1). A similar pattern of broad lytic range was reported previously, where EC.W2-6 effectively lysed up to 94.4% of clinical ST131 *E. coli* isolates and additionally demonstrated cross-species activity against *Salmonella enterica* [6,17], indicating its broad lytic capacity across diverse *E. coli* lineages.

### One-step growth curve of phage EC.W2-6

The one-step growth curve (Fig. 2C) revealed a latent period of approximately 20 min, followed by a sharp rise in phage titer, indicating host cell lysis and progeny release, with a plateau around 40 min. The estimated burst size of approximately 80 PFU/infected cell reflects a moderate replication rate sufficient for effective bacterial reduction. Comparable latency and burst size values for EC.W2-6 were reported previously (10 to 20 min; ~80 PFU/cell), indicating reproducibility across different host strains [6]. A short latent period combined with a relatively high burst size is considered optimal in therapeutic phages, as it enables efficient propagation and rapid bacterial population reduction in situ [20]. The infection cycle of EC.W2-6 enables efficient host exploitation and optimal phage amplification.

### Thermal stability of phage EC.W2-6

Thermal stability is a critical parameter for the storage and application of phages under variable environmental conditions. EC.W2-6 retained high infectivity across of 4 to 60 °C, with maximal activity observed between 20 and 40 °C. However, infectivity declined significantly at ≥70 °C (Fig. 2D). This thermal profile is consistent with findings from Shamsuzzaman et al. [6], who reported that EC.W2-6 remained stable up to 60 °C and retained partial infectivity below 80 °C. While some *Podoviridae* phages are known to lose infectivity at elevated temperatures due to structural instability [21], phage EC.W2-6 displays enhanced thermal tolerance, maintaining infectivity across a broader temperature range. Comparable thermal profiles have been observed in phages targeting multidrug-resistant *E. coli*, reinforcing EC.W2-6’s potential for therapeutic use in agricultural or field-based environments where ambient temperatures may fluctuate [17].

### pH stability of phage EC.W2-6 and implications for gastrointestinal application

Phage EC.W2-6 showed stable pH tolerance with a broad pH range, maintained a high viability between pH 5 and 8 (Fig. 2E). Phage titers declined significantly at pH 2 and 10, suggesting sensitivity to extreme acidic and alkaline conditions. Partial stability at pH 3 and 9 indicates limited tolerance to suboptimal conditions, aligning with previous observations that phage viability typically declines below pH 4 or above pH 9 [6]. Although phage EC.W2-6 demonstrates stability across a broad pH range, its exposure to the highly acidic gastric environment (pH 2 to 3) may require buffering strategies to enhance its viability and delivery efficacy [22].

To address this limitation, EC.W2-6 was combined with bentonite clay (1% to 8%) and exposed to the same pH conditions. Bentonite significantly improved phage viability under acidic conditions, particularly at pH 3 and 4, where treated groups showed approximately 1 log PFU/ml higher titers compared to the control. This protective effect is likely due to bentonite’s high surface area and cation exchange capacity, which facilitate electrostatic interactions with phage particles and provide structural protection from pH-induced denaturation [23]. Supporting this interpretation, the phage–bentonite combination exhibited a markedly less negative ZP (−13.4 mV) compared with phage (−34.2 mV) or bentonite alone (−18.9 mV) (Table 1), consistent with partial charge neutralization and electrostatic surface association. Although ZP could not be measured under acidic conditions due to instrument limitations, the complex formation observed in neutral aqueous conditions supports a stabilizing interaction that may help reduce proton-mediated phage inactivation during acidic exposure [24]. These results demonstrate that bentonite functions as a protective excipient, enhancing the gastrointestinal stability of EC.W2-6 under physiologically relevant stress conditions. Its incorporation into phage formulations presents a promising strategy to improve oral delivery efficacy and broaden therapeutic applications against enteric pathogens.

**Table 1.** Physicochemical characterization and colloidal stability of bentonite–phage composites. All values are mean ± SD_int_ of instrument replicates (*n*_runs_ ≥ 12).

Sample	Bentonite concentration (%)	Zeta potential (mV ± SD_int_)	*Z* average size (nm)	PDI
Phage (1 × 10^8^ PFU/ml)	0	−34.2 ± 6.76	623.3	0.427
Bentonite	8%	−18.9 ± 7.23	281.0	0.312
1% Combination	1%	−27.9 ± 11.4	483.4	0.540
2% Combination	2%	−26.9 ± 6.48	261.1	0.537
4% Combination	4%	−25.9 ± 6.76	429.0	0.229
8% Combination	8%	−13.4 ± 5.65	293.0	0.346

Simulated intestinal fluid (SIF) assays confirmed the protective effect of bentonite on phage stability against bile salts and enzymes (Fig. S1). EC.W2-6 alone exhibited 87.95% ± 5.31% and 61.40% ± 5.62% survival in SIF after 3 and 6 h of exposure, respectively. In the presence of 8% bentonite, viability increased to 94.24% ± 8.21% at 3 h and 70.99% ± 4.21% at 6 h; however, these differences were not statistically significant (*P* = 0.32 and *P* = 0.058, respectively) when compared to the control group. Although not significant, the upward trend suggests a potential stabilizing effect that may need further investigation with greater replication. Similar modest protective trends have been reported for clay-based excipients such as bentonite, montmorillonite, and kaolin, which can provide partial physical shielding for phages in harsh gastrointestinal environments, supporting their potential use as formulation aids for oral phage delivery [25].

### Bentonite-mediated adsorption and recovery of enterotoxins

**Fig. 3.**
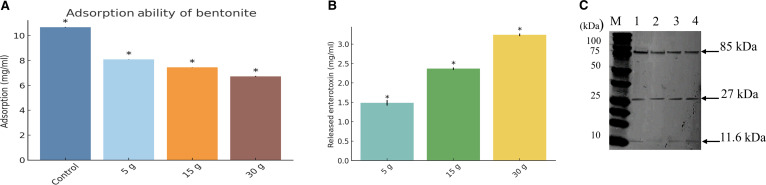
Adsorption and release of enterotoxins from *E. coli* H10407 by bentonite. (A) Adsorption efficiency of bentonite (5, 15, and 30 g) in reducing enterotoxin and outer membrane vesicle (OMV) levels from culture supernatants. (B) Recovery of enterotoxins from bentonite sediments following acidic glycine treatment (pH 2.5), showing a concentration-dependent release pattern. (C) Western blot analysis of heat-labile toxin (LT) released from bentonite. Lane M: protein marker; Lane 1: control without bentonite; Lanes 2 to 4: bentonite-treated samples (5, 15, and 30 g, respectively). Bands at 85 kDa (LT holotoxin), 27 kDa (A subunit), and 11 kDa (B subunit) confirm LT binding and release. Data are expressed as mean ± SD; *P* < 0.05 by Welch’s *t* test.

The ability of bentonite to adsorb enterotoxins and OMVs secreted by ETEC H10407 was evaluated across varying concentrations. A dose-dependent reduction in detectable toxins was observed, with all bentonite-treated groups showing significantly lower toxin levels than the control (*P* < 0.05, Welch’s *t* test; Fig. 3A). This suggests that bentonite may function as a bio-inert sorbent capable of removing macromolecular bacterial toxins from the extracellular milieu. The primary mechanism of adsorption may involve electrostatic interactions between the negatively charged silicate layers of bentonite and the cationic domains of enterotoxins. Beyond charge-based attraction, secondary forces such as van der Waals interactions, hydrogen bonding, and hydrophobic effects may synergistically stabilize the toxin–clay complexes [26]. However, the strength of these interactions may vary depending on environmental factors including pH, ionic strength, and the presence of competing molecules.

To further evaluate the binding dynamics, adsorbed toxins were desorbed from bentonite pellets using acidic glycine buffer. The 30-g treatment group showed a significant toxin recovery of approximately 3 mg/ml (*P* < 0.05; Fig. 3B), indicating that increased bentonite concentration enhances adsorption through greater surface area and a higher density of active binding sites. The observed toxin release under acidic conditions likely results from the disruption of ionic and electrostatic interactions involved in bentonite–toxin binding, caused by protonation of negatively charged surface groups at low pH. Previous studies have shown that low pH can dissociate bound molecules by weakening binding affinity, facilitating desorption in other molecular complexes, including protein–ligand interactions [24]. Although bentonite is unlikely to interact with toxins in the stomach, acidic conditions may temporarily reduce its capturing capability, bentonite is expected to regain its binding capacity in the small intestine (pH 6.5 to 7.5), enabling readsorption and sustained detoxification along the gastrointestinal tract.

Western blot analysis confirmed the identity and structural integrity of the recovered heat-labile enterotoxin (LT) secreted by ETEC H10407 following bentonite treatment (Fig. 3C). Three distinct bands were detected at approximately 85 kDa (LT holotoxin complex), 27 kDa (A subunit), and 11.6 kDa (B subunit), aligning closely with previously reported molecular weights of LT components [2]. The detection of these bands in all tested samples, including the control, indicates that LT remained structurally intact throughout the adsorption and desorption processes. LT has been shown to retain its structural integrity and biological activity when associated with ETEC-derived OMVs, indicating that the toxin is stable under adsorption and desorption conditions [27]. Furthermore, the concurrent detection of both A and B subunits implies that bentonite interacts with the holotoxin complex rather than binding selectively to individual subunits. Similar findings have demonstrated that vesicle-associated LT is delivered to host cells in a functionally active and structurally conserved form [28]. The intact protein profile suggests that bentonite does not degrade or chemically alter LT, consistent with the nondestructive binding mechanism previously reported for inert adsorbents such as smectites and activated charcoal [26].

### Endotoxin removal by bentonite

**Fig. 4.**
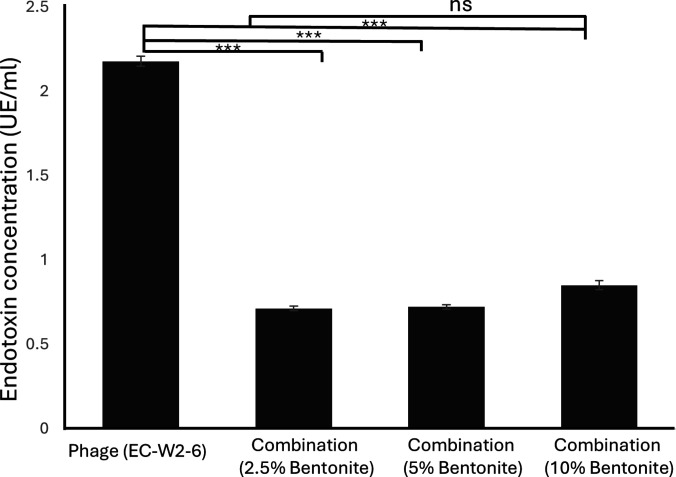
Endotoxin removal efficiency of bentonite in bacteriophage lysate. Endotoxin concentration measured in *E. coli* phage lysate after treatment with bentonite. Bars represent the endotoxin levels (EU/ml) in the untreated lysate (Control) and the lysate treated with 2.5%, 5%, and 10% bentonite. The data represent mean ± SD of the concentration of endotoxin. The experiment was performed in triplicate. ***P < 0.001 compared to EC-W2-6, while ns indicates no significant difference between the 2.5%, 5%, and 10% groups.

To determine whether bentonite reduces endotoxin levels in phage preparations, endotoxin concentrations were measured after treatment with 2.5%, 5%, and 10% bentonite. The untreated phage lysate contained high endotoxin levels (2.18 EU/ml). All bentonite concentrations significantly reduced endotoxin levels. The 2.5% and 5% treatments lowered endotoxin to 0.71 and 0.72 EU/ml, respectively, while 10% bentonite resulted in a slightly higher residual level (0.85 EU/ml). Compared with the control, 2.5% and 5% bentonite achieved maximal endotoxin clearance (~67%), whereas increasing the dose to 10% did not enhance removal beyond ~61%. No statistically significant differences were detected among the 3 bentonite concentrations, indicating that relatively low bentonite levels are sufficient to achieve substantial endotoxin adsorption (Fig. 4). Importantly, endotoxin removal did not appear to adversely affect phage functionality, as lytic activity remained comparable between bentonite-treated and untreated phage preparations (Fig. S2).

However, these findings are consistent with previous reports demonstrating that clay minerals, including bentonite, can effectively bind LPS. Prior studies have shown that bentonite exhibits strong endotoxin adsorption capacity in vitro and within gastrointestinal environments, resulting in reduced endotoxin levels in animal models [29]. Additional investigations have similarly reported efficient adsorption of *E. coli*-derived LPS by bentonite and related clay minerals in aqueous and simulated intestinal systems, as assessed by the Limulus amebocyte lysate assay [14]. Collectively, these observations align well with the endotoxin reduction observed in the present study.

### Bentonite-mediated reduction of nanoparticle concentration and size distribution

**Fig. 5.**
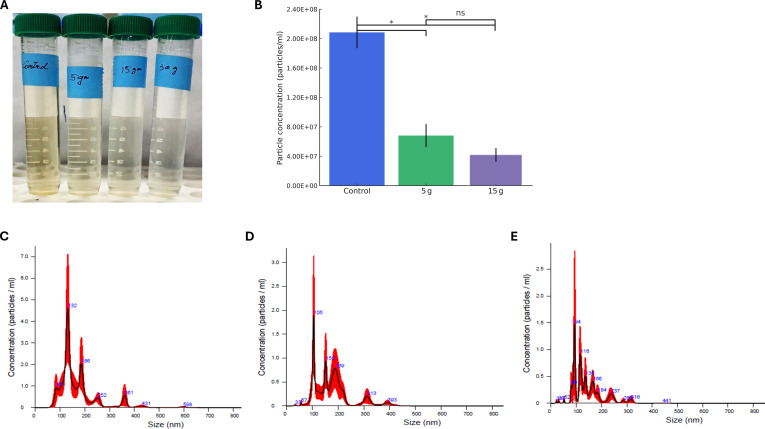
Nanoparticle tracking analysis (NTA) and visual assessment of outer membrane vesicles (OMVs) following bentonite treatment. (A) Visual appearance of nanoparticle suspensions treated with 0, 5, 15, and 30 g of bentonite. The control sample appears turbid, while turbidity decreases progressively with increasing bentonite concentration. (B) OMV concentrations were quantified by NTA. The 30-g group was excluded due to inconsistent detection, likely caused by particle aggregation or instrument saturation. (C to E) Particle size distribution profiles of the control, 5-g, and 15-g groups, respectively. Bentonite treatment resulted in lower particle concentrations and a shift toward smaller particle sizes. Data are expressed as mean ± SD (*n* = 3 for control and 5 g; *n* = 2 for 15 g). Statistical analysis was performed using unpaired 2-tailed *t* tests; *P* < 0.05 was considered significant (*), and *P* ≥ 0.05 not significant (ns).

The effect of bentonite on reducing particle concentration and size distribution was evaluated to understand its role in toxin and vesicle removal. Visually, a progressive decrease in turbidity was observed with increasing bentonite concentrations (Fig. 5A). The control sample remained visibly turbid due to a high nanoparticle load, whereas 5- and 15-g treatments exhibited decreased turbidity. The 30-g group appeared nearly transparent, suggesting extensive aggregation or sedimentation, which was corroborated by inconsistent NTA detection.

NTA confirmed a dose-dependent reduction in vesicle-like particles (Fig. 5B). The control group exhibited 1.95 × 10^8^ particles/ml, which declined to 6.81 × 10^7^ and 4.11 × 10^7^ particles/ml after treatment with 5 and 15 g of bentonite, respectively. These findings support bentonite’s efficacy in removing extracellular vesicles, likely through mechanisms involving aggregation and sedimentation [30]. Beyond reducing vesicle abundance, bentonite also altered the particle size distribution (Fig. 5C to E). The control exhibited a broad, multimodal profile with peaks between 100 and 200 nm, consistent with the typical size range of *E. coli*-derived OMVs [31]. In contrast, the 5- and 15-g treatments resulted in narrower size distributions and reduced particle abundance, indicating that bentonite effectively removes vesicles across different size ranges.

In the 30-g group, NTA detection was inconsistent, likely due to extensive aggregation and sedimentation. Bentonite’s negatively charged silicate layers facilitate electrostatic interactions with nanoparticles, enhancing their destabilization and removal from suspension at higher concentrations [12]. Such interactions may reduce Brownian motion and shift particles beyond the NTA detection range or promote sedimentation, rendering them undetectable [32]. Additionally, bentonite may absorb surface-associated biomolecules such as proteins or polyphenols, thereby disrupting colloidal stability. Due to this inconsistency, data from the 30-g group were excluded from quantitative comparisons to maintain clarity in the dose–response trend.

The concurrent analysis of NTA particle count reduction and ZP shifts indicates that bentonite removes extracellular nanoparticles through 2 coexisting but mechanistically distinct processes. First, macromolecular toxins can undergo true adsorption onto bentonite surfaces through electrostatic and secondary interactions, consistent with the toxin-binding results. Second, for colloidal particles such as OMVs, bentonite primarily induces destabilizing heteroaggregation rather than classical adsorption. Because both bentonite and OMVs are predominantly anionic, charge modification reduces their electrostatic repulsion and promotes aggregation, leading to gravitational sedimentation as the dominant clearance pathway. Colloidal systems are normally stabilized by surface charge; however, reductions in ZP disrupt this stability, promoting aggregation and flocculation. As aggregates increase in hydrodynamic diameter and effective density, they sediment rapidly—a bulk removal process distinct from true adsorption, in which bound particles typically remain dispersed. This sedimentation-driven mechanism is well established in clay–colloid systems and explains the dose-dependent decreases in particle counts, turbidity, and NTA-detectable vesicles observed in bentonite-treated samples [33].

**Fig. 6.**
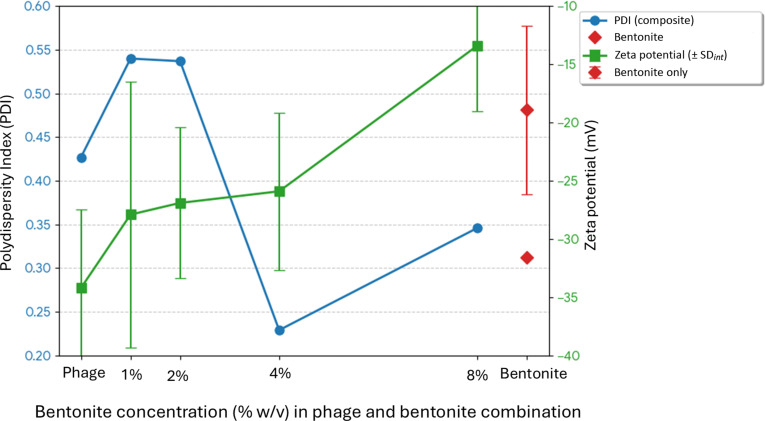
Concentration-dependent colloidal stability and surface charge of bentonite–phage complexes. The figure tracks the polydispersity index (PDI) (left axis) and zeta potential (ZP) (right axis) against phage and bentonite combination with 1%, 2%, 4%, and 8% concentration (% w/v) of bentonite. Error bars represent the internal instrument standard deviation (SD_int_) from *n*_runs_ ≥ 12. The phage and the isolated bentonite are plotted as reference.

ZP analysis of phage EC.W2-6 showed a baseline value of −34.2 mV, indicating a strongly negative surface charge. Increasing bentonite concentrations progressively shifted the ZP toward less negative values, reaching −13.4 mV at the 8% combination, consistent with electrostatic association between bentonite and phage that may enhance phage stability in gastrointestinal conditions [34]. This shift was not monotonic; the 1% combination exhibited the highest charge heterogeneity (SD_int_ = 11.4 mV), aligning with elevated polydispersity index (PDI) values and indicating maximal colloidal instability at low clay concentrations (Fig. 6). Size analysis showed an apparent reduction in hydrodynamic size—from 623.3 nm (phage alone) to 293.0 nm at the 8% combination (Table). This size reduction is unlikely to reflect phage–phage aggregation, but rather the formation of compact phage–bentonite complexes that behave as smaller, denser hybrid particles in suspension. This interpretation is supported by the preservation of phage lytic activity in all bentonite-treated groups (Fig. S2), supporting the interpretation that bentonite does not induce detrimental phage self-aggregation.

In contrast, PDI analysis further revealed that the 4% combination produced the most uniform particle size distribution (PDI = 0.229), whereas the 1% and 2% combinations exhibited high polydispersity (PDI = 0.540 and 0.537, respectively) (Table). Thus, while the 8% combination induced the most substantial reduction in hydrodynamic size, the 4% combination produced the most uniform suspension. These observations suggest that compaction and uniformity are optimized at different bentonite concentrations, reflecting distinct physicochemical interactions between bentonite and the phage. Uniform particle distributions are generally considered advantageous for oral delivery, as consistent hydrodynamic behavior enhances gastrointestinal transit, diffusion, and bioavailability in nanoparticle-based systems. Phage stability has likewise been associated with reduced aggregation and consistent particle size, supporting improved therapeutic performance during oral administration [35]. By promoting charge modification and structural reorganization across varying concentrations, bentonite may therefore contribute to enhanced phage stability and increase the likelihood of viable phage particles reaching the intestinal target site, potentially improving therapeutic efficacy. Further evidence of reduced particle abundance was provided by NTA-derived scatter intensity plots (Fig. S3), which showed a progressive decline in signal intensity with increasing bentonite concentration. This observation was visually supported by real-time NTA video recordings (Movie S1), which demonstrated lower vesicle density and diminished particle movement in the 5- and 15-g groups compared to the control.

Overall, these findings indicate that bentonite reduces nanoparticle abundance via dose-dependent aggregation and sedimentation, likely through surface charge neutralization and destabilization of colloidal structure. The observed trend aligns with previous findings indicating that nanoparticle aggregation particularly under high ionic or polymeric stress can hinder NTA detection by reducing Brownian motion or promoting particle fusion [32]. Future research should elucidate bentonite’s molecular-level interactions with large vesicles to optimize its utility in vesicle-targeted detoxification strategies.

### In vivo protection against ETEC H10407 infection by bentonite and phage EC.W2-6

**Fig. 7.**
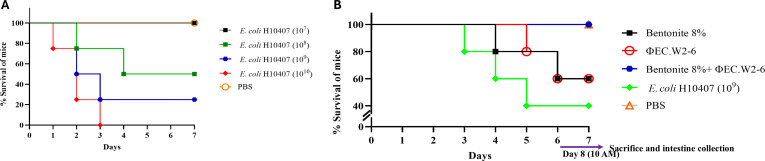
Survival analysis following *E. coli* H10407 infection and therapeutic intervention in a murine model. (A) Determination of the median lethal dose (LD_50_) of *E. coli* H10407. Mice (*n* = 4/group) were orally administered varying doses (10^7^ to 10^10^ CFU/mouse) of *E. coli* H10407. Survival was monitored for 7 days. The LD_50_ was estimated at approximately 10^8^ CFU/mouse. (B) Survival outcomes of infected mice following treatment. Mice (*n* = 5/group) were infected with *E. coli* H10407 (10^8^ CFU/mouse) and treated with 8% bentonite, phage EC.W2-6, and a combination of both. Control groups included PBS and infection-only mice. Survival was recorded daily for 7 days. Surviving animals were sacrificed on day 8 (10:00 AM) for intestinal sample collection and microbiome analysis.

A murine survival assay was conducted to assess the protective efficacy of bentonite and phage EC.W2-6 against ETEC H10407-induced diarrhea. To determine the dose-dependent pathogenicity, mice were orally administered different doses of ETEC H10407 ranging from 10^7^ to 10^10^ CFU per mouse. Mice administered 10^9^ or 10^10^ CFU exhibited complete mortality by day 3, whereas the 10^7^ CFU group showed moderate lethality (Fig. 7A). Based on these outcomes, the median lethal dose (LD₅₀) was estimated at approximately 10^8^ CFU/mouse and was selected for subsequent therapeutic assessments, consistent with previously reported murine models of ETEC infection [2,4].

An optimal bentonite concentration of 8% was determined based on enhanced survival and attenuation of clinical signs associated with ETEC-induced diarrhea despite ZP and PDI analyses indicating that 4% bentonite produced a more uniform particle size distribution. Single therapy with either 8% bentonite or phage EC.W2-6 (MOI 0.1) conferred partial protection, with survival rates of 60%, whereas the infection-only group exhibited 40% survival. Notably, combination therapy of bentonite and EC.W2-6 provided complete protection with 100% survival (Fig. 7B) suggestive of a synergistic therapeutic effect. To evaluate diarrheal severity, representative fecal samples were visually assessed throughout the experiment period. Visual inspection revealed distinct differences in stool consistency among groups: healthy mice (PBS control) produced well-formed, pellet-like feces, while infected mice presented varying degrees of watery stools, indicative of intestinal dysfunction and fluid imbalance (Fig. S4). These clinical findings were congruent with survival outcomes, supporting a correlation between treatment efficacy and disease severity [36].

However, the enhanced protection observed in the combination group may result from the complementary mechanisms of bentonite and phage EC.W2-6. Bentonite binds bacterial macromolecules such as heat-labile enterotoxins (LT), thereby reducing toxin bioavailability and mitigating intestinal inflammation [13]. Concurrently, EC.W2-6 selectively infects and lyses ETEC H10407, reducing bacterial load and limiting disease progression. The convergence of these mechanisms provides both bacterial clearance and toxin neutralization, leading to improved therapeutic efficacy and host survival. Similar synergistic phage-adsorbent strategies have been reported in other infectious disease models, where phages combined with adjunct agents enhanced survival and attenuated inflammation [6].

In the present experiment, we did not directly assess pro-inflammatory cytokine production or anti-phage IgG responses. However, our recently published study employing the same phage, EC.W2-6, examined these immune parameters in both infected and noninfected mouse models. In that study, serum collected after the initial administration (Day 0) and again 10 days later demonstrated a significant reduction in systemic cytokines, including IL-6, IFN-γ, TNF-α, and IL-1β, following EC.W2-6 treatment compared with pathogen-only controls. Importantly, EC.W2-6 was also shown to induce a markedly lower anti-phage IgG response over time, indicating a favorable immunological profile with reduced systemic reactivity. Together, these findings support the immunological safety of EC.W2-6 and are consistent with its therapeutic activity observed in the current study [37].

While the observed protection of mice indicates effective therapeutic delivery, a comprehensive in vivo characterization of the combined system’s pharmacokinetics and biodistribution will be essential for full translational validation. In particular, the survival, replication, and tissue distribution of phages following coadministration with bentonite were not evaluated in this short-term efficacy study. Nevertheless, supportive in vitro acidic stress assays (pH 3 and 4) demonstrated that bentonite improves EC.W2-6 viability under low-pH conditions, and physicochemical analyses are consistent with a stabilizing bentonite–phage association that may contribute to improved oral delivery (Table and Fig. 6). Similarly, the assessment of systemic exposure and host immune activation, such as phage-specific IgG/IgA or inflammatory cytokine profiles, remains a critical priority to fully define translational safety and exclude unintended immunogenicity. Future investigations are therefore prioritized to longitudinally quantify viable phage titers, evaluate systemic exposure, and characterize host immune responses in the ETEC diarrhea cohort.

### Microbial community shifts post-ETEC infection and therapeutic intervention

All microbial analyses were conducted using intestinal samples collected for 7 days post-treatment, demonstrating the potential for microbiome recovery within a short time frame.

### Alpha-diversity analysis

**Fig. 8.**
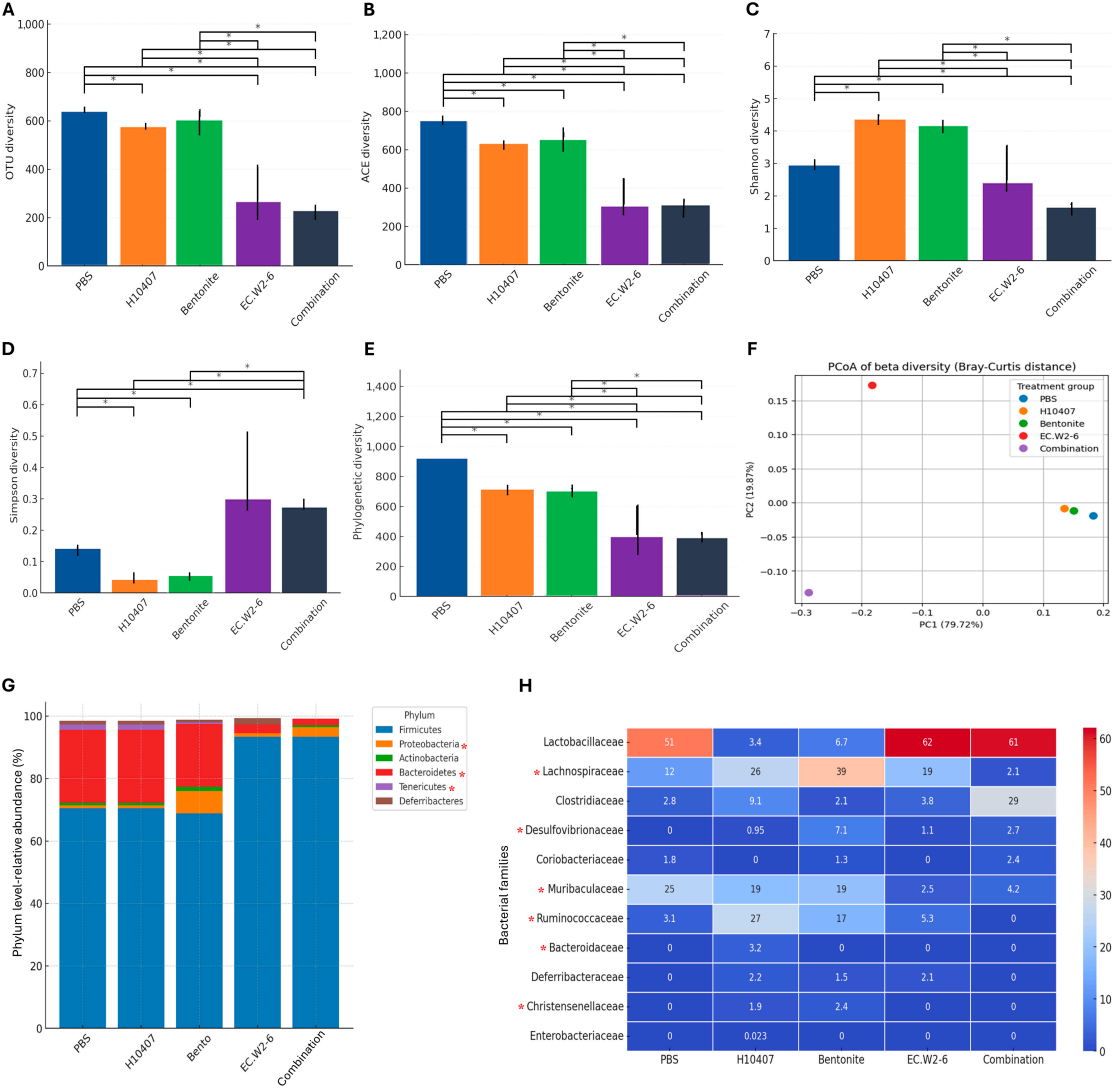
Effects of therapeutic treatments on gut microbiota diversity and composition in ETEC-infected mice. (A to E) Alpha diversity indices across 5 treatment groups (PBS, H10407, bentonite, EC.W2-6, and Combination) based on observed OTUs (A), ACE (B), Shannon (C), Simpson (D), and phylogenetic diversity (E). Data are presented as mean ± SD. Statistical significance between groups was determined using pairwise Wilcoxon rank-sum tests (*P* < 0.05). (F) The PCoA plot visualizes the overall distinct shifts between the 5 treatment groups. The clustering indicates a significant separation of the microbial communities, which was statistically confirmed by both PERMANOVA (pseudo-*F* = 6.220, *P* = 0.012) and the nonparametric Kruskal–Wallis *H* test (*H* = 11.40, *P* = 0.0224). PC1 and PC2 explain 79.72% and 19.87% of the total variation, respectively. (G) Overall differences in phylum community structure were assessed by PERMANOVA (*P* = 0.001). Differences in individual phylum abundance were determined by the Kruskal–Wallis *H* test with subsequent Dunn’s post-hoc pairwise comparisons. All *P* values were adjusted for multiple comparisons using the false discovery rate (FDR) method, with significance set at *P*_adj_ < 0.05. An asterisk (*) placed next to a phylum name in the figure denotes that its relative abundance was significantly different across the treatment groups (*P*_adj_ < 0.05). (H) Heatmap showing the median relative abundance (%) of key bacterial families across the 5 experimental groups. An asterisk (*) indicates that the family showed significant overall differential abundance by the Kruskal–Wallis test (FDR-corrected, *Q* < 0.20).

Alpha-diversity indices (Fig. 8A to E) showed that the PBS control group had the highest microbial richness and evenness, reflective of a stable gut ecosystem. In contrast, ETEC H10407 infection significantly reduced diversity (*P* < 0.01), indicative of pathogen-induced dysbiosis and intestinal inflammation [4,38]. Bentonite treatment partially recovered this effect, improving ACE and phylogenetic diversity, suggesting its role in neutralizing bacterial toxins and promoting early stabilization of gut microbiota [39]. Phage EC.W2-6 alone altered microbial composition and reduced overall diversity, possibly due to selective pressure on susceptible taxa. Notably, the combination treatment significantly increased Shannon and Simpson indices (*P* < 0.05), indicating improved microbial richness and evenness through the synergistic interaction of bentonite and phage EC.W2-6 [40].

### Beta-diversity and microbial community structure

Principal coordinate analysis (PCoA) utilizing Bray–Curtis dissimilarity metrics (Fig. 8F) revealed distinct clustering of gut microbial communities among treatment groups. This overall difference in community structure was statistically significant, as supported by PERMANOVA (pseudo-*F* = 6.22, *P* = 0.012) and confirmed by the nonparametric Kruskal–Wallis *H* test (*P* = 0.0224 for the single measured variable). PBS controls formed a tight cluster, representing a stable microbiota, while the ETEC-infected group diverged significantly, reflecting pathogen-induced dysbiosis as previously described in enteric infection models [40]. Bentonite-treated samples shifted toward the PBS cluster, suggesting partial shift toward microbial homeostasis, likely through toxin adsorption and reduction of intestinal inflammation [41]. In contrast, the EC.W2-6 treated group displayed a distinct compositional shift, indicative of phage-mediated modulation of specific bacterial populations [42]. The combination therapy group formed an intermediate cluster between bentonite and PBS, implying that bentonite’s stabilizing effect mitigates phage-induced disruption, facilitating microbiota reassembly toward a healthier profile [38].

### Phylum-level taxonomic comparison

Phylum-level analysis (Fig. 8G) revealed clear microbial shifts following ETEC challenge and treatment, with PERMANOVA indicating a highly significant overall community difference among groups (pseudo-*F* = 21.79; *P* = 0.001). Kruskal–Wallis testing with FDR correction confirmed significant alterations in *Proteobacteria*, *Bacteroidetes*, and *Tenericutes* (*Q* = 0.033). The PBS group maintained a stable *Firmicutes–Bacteroidetes* balance reflective of healthy microbial homeostasis [43]. ETEC H10407 infection caused a visually apparent expansion in *Proteobacteria* (nonsignificant vs. PBS; *P* = 0.508), accompanied by significant reductions in *Bacteroidetes* (adjusted *P* = 0.026 vs. Combination) and *Firmicutes*, and a significant elevation in *Tenericutes* (*P* = 0.019 vs. PBS). This dysbiosis is consistent with infection-driven oxidative disruption, epithelial injury, and redox shifts that favor Enterobacteriaceae expansion [38,44].

Dunn’s post-hoc comparisons revealed distinct recovery profiles among treatments. Bentonite significantly reduced *Proteobacteria* relative to the H10407-infected group (*P* = 0.022) and restored its abundance to levels not significantly different from PBS (adjusted *p* = 1.000). Bentonite also promoted the early recovery of *Bacteroidetes* to values not significantly different from healthy controls (*P* = 0.237). Phage EC.W2-6 similarly suppressed *Proteobacteria* (*P* = 0.025 vs. H10407), but *Bacteroidetes* remained partially depressed, indicating that pathogen clearance may be insufficient to fully reestablish phylum-level equilibrium during the acute phase.

Notably, the combination therapy demonstrated the most consistent phylum-level recovery profile observed at the 7-day time point, with no significant differences from PBS across all perturbed phyla (*P* > 0.08), suggesting cooperative microbiota recovery rather than simple additive effects. These patterns support a dual-action interaction wherein bentonite’s known enterotoxin-binding capacity reduces the dysbiotic burden, thereby facilitating more effective phage-mediated pathogen clearance [45–47]. The observed shift in *Firmicutes–Bacteroidetes* ratios further indicates the recovery of mucosal nutrient cycling and short-chain fatty acid (SCFA) metabolism. Together, these changes support the restoration of intestinal epithelial homeostatic regulation and reinforce the idea that bentonite–phage coadministration surpasses either single therapy in reversing ETEC-induced dysbiosis and promoting early reassembly of microbial homeostasis [46,48]. While the short-term phylum-level restoration suggests a robust shift toward a homeostatic state following coadministration of phage and bentonite, assessing the long-term stability and functional persistence of this restored microbiota remains a critical priority for future in vivo studies.

### Family-level microbiome dynamics

Family-level microbiota profiling (Fig. 8H) revealed significant treatment-dependent restructuring of gut communities, supported by PERMANOVA analysis (Pseudo-*F* = 13.75, *P* = 0.001), indicating that treatment effects contributed more than interindividual variability. Kruskal–Wallis testing with FDR correction identified 11 differentially abundant families (*Q* < 0.20), 6 of which—*Bacteroidaceae, Ruminococcaceae, Desulfovibrionaceae, Muribaculaceae, Christensenellaceae*, and *Lachnospiraceae*—remained visually distinct across groups and exhibited significant differences in Dunn post-hoc comparisons (nominal *P* < 0.05).

In PBS control, microbiota was dominated by beneficial SCFA-producing families such as *Lactobacillaceae* (51%), *Lachnospiraceae* (12%), *Muribaculaceae* (25%), and *Ruminococcaceae* (3.1%), consistent with a stable and homeostatic intestinal environment [43]. Infection with *E. coli* H10407 induced a statistically validated dysbiosis characterized by significant enrichment of *Bacteroidaceae* (*Q* = 0.0387), with higher median abundance in infected mice compared with all treatment groups (*P* ≤ 0.0083). Increases in *Clostridiaceae* (9.1%) and *Enterobacteriaceae* (0.023%) further reflected an inflammation-associated microbial profile [46].

Bentonite treatment induced a distinct restructuring of the microbial community. *Lachnospiraceae* was markedly enriched (39%; *Q* = 0.0638), significantly exceeding levels in the combination group (*P* = 0.0349). Bentonite also increased abundance of *Desulfovibrionaceae* (*Q* = 0.0395; 7.1%) relative to PBS controls (*P* = 0.0216). Although this family is associated with hydrogen sulfide production and intestinal inflammation, its abundance remained below levels generally considered nonharmful (<10%) [47]. In addition, *Christensenellaceae* was selectively enriched following bentonite treatment (2.4%; *Q* = 0.0387) compared with phage-only, combination, and PBS groups (*P* = 0.0334), indicating a treatment-specific modulatory effect.

Phage only treatment led to a strong recovery of *Lactobacillaceae* (62%), effectively reversing the infection-induced decline in this key beneficial family. However, *Muribaculaceae* (2.5%) and *Clostridiaceae* (3.8%) remained significantly reduced relative to PBS controls (*P* = 0.0337), indicating that phage-mediated pathogen clearance alone does not fully restore commensal diversity during the acute phase [48]. Notably, combination therapy showed the most extensive early microbiota restoration within the 7-day period. *Lactobacillaceae* (61%) and *Lachnospiraceae* (2.1%) approached PBS-like levels while *Muribaculaceae* (4.2%) exhibited partial recovery (*P* = 0.0394 vs. PBS), indicating a gradual resurgence of beneficial taxa. These results suggest that within a short period, cotreatment not only suppresses pathogen-associated taxa but also associated with the early return of SCFA-producing families critical for intestinal stability and host health. However, the abundance of *Clostridiaceae* (29%) suggests potential microbial competition or transitional restructuring following treatment [43,45]. Importantly, *Enterobacteriaceae* was completely eliminated in the combination, phage-only, and bentonite-only groups, indicating the effective suppression of this inflammation-associated family across all treatment modalities [49].

Collectively, these findings highlight the therapeutic potential of combining toxin-adsorbing clays with targeted phage therapy as a strategy for mitigating enteric infection-associated dysbiosis. Bentonite contributes to toxin neutralization and microbiota stabilization, while phage EC.W2-6 facilitates pathogen-specific clearance without disrupting beneficial taxa [50]. This dual intervention could reduce reliance on conventional antibiotics and facilitate microbiome recovery within the short period. However, further studies are required to assess long-term microbial stability, functional metabolic restoration, and host–microbiota interactions during extended recovery following phage–bentonite coadministration.

## Conclusion

This study introduces a dual-action therapeutic strategy combining phage EC.W2-6 with bentonite clay to treat ETEC H10407-induced diarrhea and intestinal dysbiosis. EC.W2-6 provides rapid and specific bacterial lysis, while bentonite simultaneously adsorbs macromolecular toxins—including LT, LPS, and OMVs—and acts as a protective excipient that enhances phage stability under gastrointestinal conditions. Together, these mechanisms reduce pathogen burden, mitigate toxin-mediated intestinal damage, and promote early reassembly of microbial diversity. The combination therapy achieved complete survival in the murine model and promoted early restoration of a gut microbial structure resembling healthy controls, outperforming either single therapy. These findings support the potential of phage–bentonite therapy as a microbiome-focused, nonantibiotic intervention for enteric infections and provide a foundation for future translational development.

## Data Availability

All data generated or analyzed during this study are included in this article. The GenBank accession number for phage EC.W2-6 is PP445229.
