# Supplementary material for: Bentonite-Based Functional Nanoclay Enhances Bacteriophage Therapy against Enteric Infections via Toxin Adsorption and Microbiome Recovery
Source: Biomater Res. 2026 Jan 29;30:0310. doi: 10.34133/bmr.0310 (PMC12852568; doi:10.34133/bmr.0310)
Supplement: Supplementary 1 — Figs. S1 to S4 Table S1 Movie S1 [file bmr.0310.f1.zip › Supplementary data.docx]

Title: Bentonite-Based Functional Nanoclay Enhances Bacteriophage Therapy Against Enteric Infections via Toxin Adsorption and Microbiome Recovery

Supplementary data


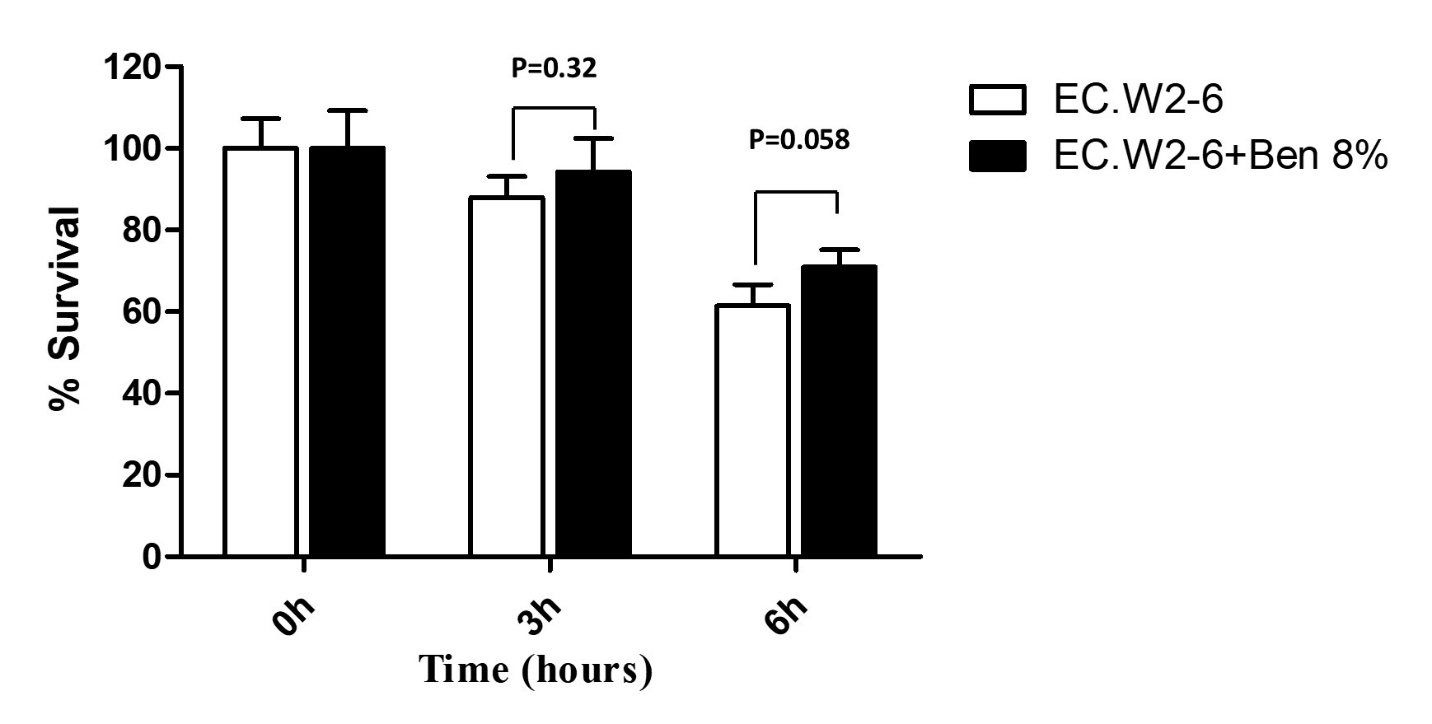


Figure S1: Protective effect of bentonite on phage viability in simulated intestinal fluid. Survival (%) of Phage EC.W2-6 with and without 8% (w/v) bentonite over 6 h exposure in simulated intestinal fluid (SIF). Phage viability is expressed relative to initial PFU/mL. Data presented as mean ± SD (*n*=3).

.


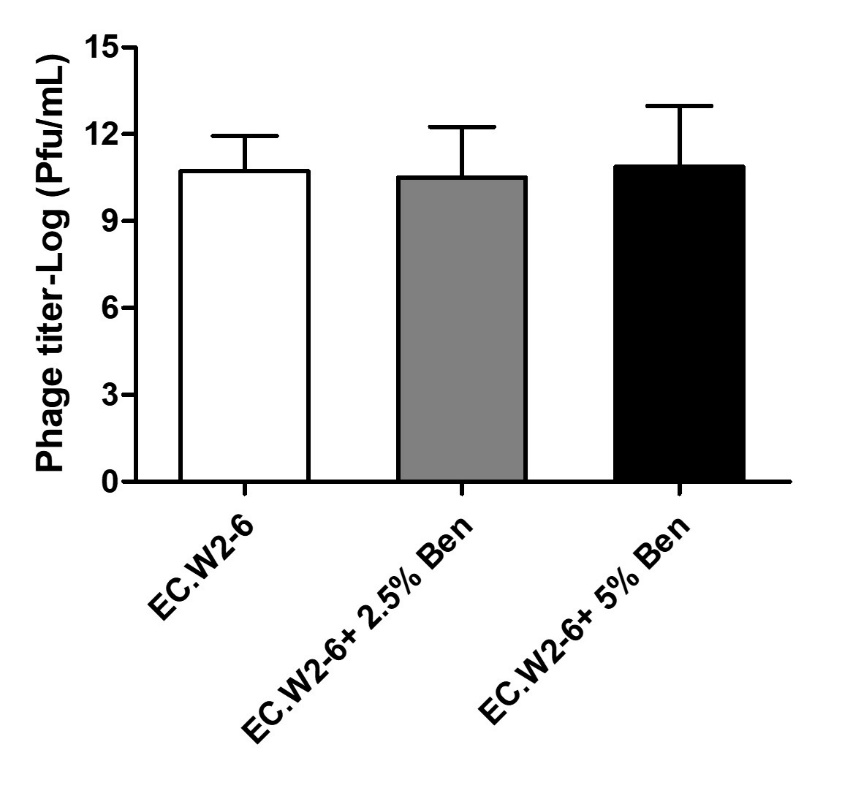


**Figure S2.** Compatibility of phage EC.W2-6 with bentonite. Phage EC.W2-6 (10¹⁰ PFU/mL) was co-incubated with increasing concentrations of bentonite (2.5%, 5% and 10%) for 1 hour at 37 °C. Plaque assay results demonstrated no significant difference in phage titer across the treatment groups (*p* > 0.05), confirming that bentonite does not negatively interfere with phage viability under physiological conditions.


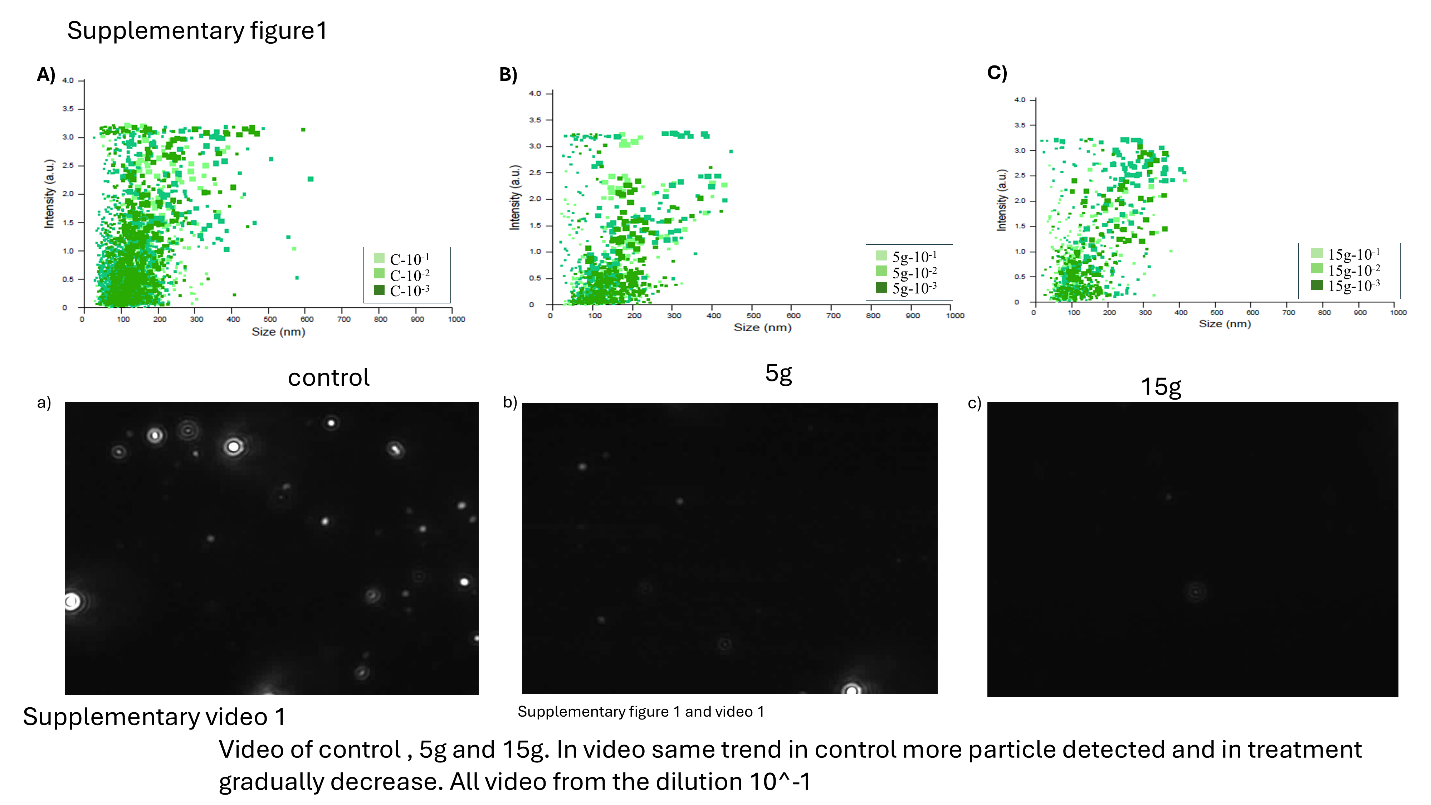


**Figure S3.** Nanoparticle tracking analysis (NTA) showing size versus intensity plots for (A) control; (B) 5 g bentonite; (C) 15 g bentonite samples across serial dilutions (10⁻¹ to 10⁻³). A progressive reduction in signal intensity was observed with increasing bentonite concentration, reflecting a dose-dependent decrease in nanoparticle abundance. Lower intensity indicates reduced particle counts and diminished light scattering efficiency.


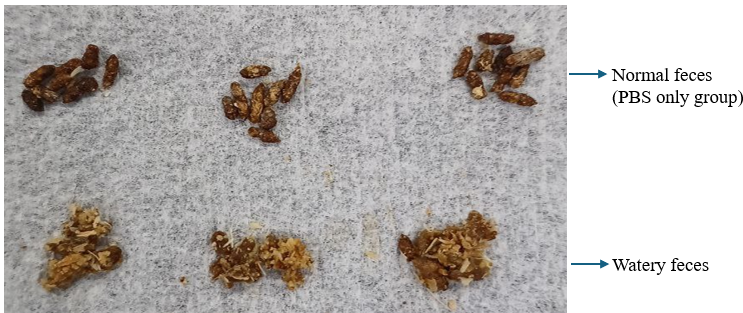


**Figure S4**. Representative fecal samples from healthy and ETEC-infected mice. Normal, well-formed feces from PBS-treated control mice (top row) contrast with watery, diarrheal feces from ETEC-infected mice (bottom row), visually indicating infection-induced gastrointestinal symptoms.

**Video S1**: Nanoparticle tracking analysis video of control, 5g, and 15g treatments.

Representative NTA video showing nanoparticle detection from diluted samples (10⁻¹ dilution). The video demonstrates a clear reduction in the number of detected particles with increasing bentonite concentration. The control sample shows the highest number of detected particles, while the 5g and 15g treatments show a progressive decrease, supporting the dose-dependent reduction trend.

**Table S1**: Efficacy of bacteriophage against ETEC strain

| Strain | Characteristics | Remark | EC.W2-6 |
| --- | --- | --- | --- |
| H10407-P | O78:H11 | LT | CL |
| PB176 | O6:H16 | LT | CL |
| H10407 | O78:H11 | LT, ST, CFA/I | CL |
| E9034/A | O8:H9 | LT, ST, CFA/2 (CS3) | SCL |
| E8775 | O25:H24 | LT, ST, CFA/2 (CS4, CS6) | CL |
| ATCC 25922 | - | - | CL |
